# Surinfection d’une endocardite de Libman-Sacks chez une patiente lupique: rapport de cas

**DOI:** 10.11604/pamj.2022.42.278.34205

**Published:** 2022-08-12

**Authors:** Nuance Divine Tchiloemba Tchibinda, Fatimatou Zahra Coulibaly, Chimène Willer Kitihoun Mamadou, Rajae Bennani, Nadia Fellat, Rokya Fellat

**Affiliations:** 1 Service de Cardiologie A, Centre Hospitalier Universitaire Ibn Sina, Université Mohammed V, Rabat, Maroc

**Keywords:** Endocardite de Libman-Sacks, endocardite non infectieuse, syndrome des antiphospholipides, lupus érythémateux disséminé, cas clinique, Libman-Sacks endocarditis, non-infectious endocarditis, antiphospholipid syndrome, systemic lupus erythematosus, case report

## Abstract

L'endocardite de Libman-Sacks constitue une manifestation cardiaque peu fréquente de la maladie lupique au cours de laquelle il existe une végétation non infectieuse au niveau des valves cardiaques. La plupart des patients sont asymptomatiques. Cependant, dans la présentation clinique, la forme aigue peut imiter celle de l'endocardite infectieuse et compliquer à la fois le diagnostic différentiel et le traitement. Nous rapportons l'observation d'une patiente de 28 ans, suivie pour lupus érythémateux depuis 2018; qui présentait des signes et symptômes compatibles avec une endocardite infectieuse, et dont les différentes explorations ont permis de conclure à une endocardite de Libman-Sacks surinfectée. l'évolution était fatale malgré le traitement associant une bi-antibiothérapie et des corticoïdes.

## Abstract (en)

Libman-Sacks endocarditis is an uncommon cardiac manifestation of lupus, characterized by noninfective heart valve vegetations. Most patients are asymptomatic. However, clinical manifestations of acute forms can mimic infectious endocarditis and complicate both differential diagnosis and treatment. We here report the case of a 28-year-old female patient with lupus erythematosus followed up from 2018. She had signs and symptoms supporting the diagnosis of infective endocarditis. Assessments allowed for the diagnosis of superinfection Libman-Sacks endocarditis. The patient died despite the combination of bi-antibiotic therapy and corticosteroids.

## Introduction

L'endocardite de Libman-Sacks (ELS), également connue sous le nom d'endocardite verruqueuse atypique, est une manifestation cardiaque bien connue du lupus érythémateux disséminé, trouvée chez un patient atteint de la maladie sur 10, chez qui des végétations stériles sont retrouvées, le plus souvent en post-mortem. Elles affectent plus fréquemment les valves du cœur gauche, en particulier le feuillet mitral postérieur [1]. La hantise, c'est la surinfection mais aussi la survenue dans un contexte de syndrome des anti-phospholipides qu'il faut toujours rechercher. À travers cette observation, nous discutons les aspects cliniques, diagnostiques, pronostiques et les options thérapeutiques que requiert l'endocardite de Libman-Sacks.

## Patient et observation

**Informations de la patiente:** madame MR âgée de 28 ans, suivie depuis 2018 pour lupus érythémateux disséminé en rémission avec arrêt du traitement d'entretien depuis 6 mois. Elle rapportait une notion d'implantation de prothèse dentaire 02 mois avant son admission; il n'y avait pas de foyer infectieux chronique individualisé, ni de cardiopathie connue. La patiente a consulté pour une fièvre persistante évoluant depuis deux semaines, associée à une polyarthralgie d'allure inflammatoire, symétrique au niveau des poignets, des genoux et des chevilles.

**Résultats cliniques:** à l'admission, elle était fébrile à 39°C, l'état hémodynamique était correct, l'auscultation cardiaque ne révélait pas de souffle; Par ailleurs, les poignets, les genoux et les chevilles étaient tuméfiés et douloureux, Il n'y avait pas de lésions cutanées évocatrices de lupus.

**Figure 1:**
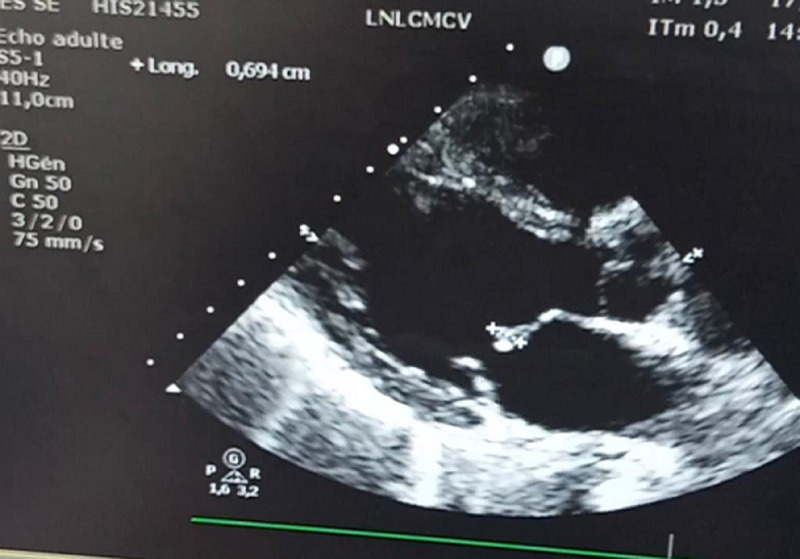
échographie transthoracique, coupe para-sternale grand axe, montrant une végétation appendue sur la grande valve mitrale mesurant 6x4 mm

**Figure 2:**
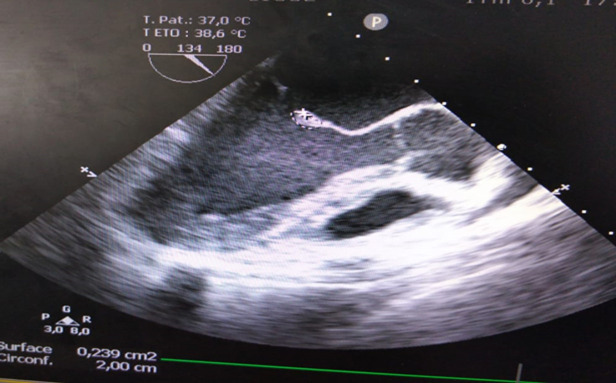
échographie transoesophagienne, à 120 degré confirmant la présence d'une végétation au niveau de la grande valve mitrale

**Démarche diagnostique:** le bilan biologique a objectivé un syndrome inflammatoire avec une protéine C reactive (CRP) élevée à 83 mg/l, ainsi qu'une leucopénie à 3400. La procalcitonine était négative. Le bilan rénal était normal et les hémocultures étaient négatives (3 séries réalisées au moment des pics fébriles et restées négatives même après 7 jours de culture). Le sérodiagnostic de certains microorganismes (ex: Coxiella burnetii, Bartonella spp, Chlamydia psittaci, Brucella spp) n'a pu être réalisé compte tenu de l'indisponibilité des milieux spéciaux au sein de notre service. Morphologiquement, l'échographie transthotacique (Figure 1) et l'échographie transoesophagienne (Figure 2) ont objectivé une végétation appendue à la grande valve mitrale mesurant 6x4mm. Devant ce tableau, le diagnostic d'endocardite infectieuse probable était alors évoqué selon les critères de Duke modifiés (un critère majeur: la végétation et un critère mineur: la fièvre).

**Intervention thérapeutique et suivi:** une antibiothérapie probabiliste par voie parentérale associant la gentamicine (3 mg/kg/jr) et la ceftriaxone (2 g/jr) a été instaurée et bien suivie. Toutefois, l'évolution clinique et biologique était stationnaire jusqu'au 7^e^ jour notamment, la persistance des douleurs articulaires, de la fièvre et du syndrome inflammatoire biologique. Ceci avait motivé la réalisation d'un bilan immunologique qui a montré un titre élevé des anticorps anti-nucléaire à 640 UI par ml, des anticorps anti-ADN natifs positifs à 162 UI par ml; témoignant de la réactivation de sa maladie lupique. Devant ce tableau, le diagnostic d'endocardite de Libman-Sacks surinfectée avait été retenu; motivant ainsi l'adjonction par les internistes d'une corticothérapie à 1 mg/kg/jour à l'antibiothérapie.

Par ailleurs, les anticorps antiphospholipides notamment les anticardiolipines étaient positifs (IgG à 120UGPL) évoquant un contexte de syndrome des anticorps anti-phospholipides. Toutefois les résultats nous sont parvenus tardivement, d'où l'absence d'anticoagulation dans le protocole thérapeutique. L'évolution sous ce protocole (antibiotiques + corticothérapie) a été marquée par la survenue d'un sepsis sévère compliqué d'une coagulation intravasculaire disséminée entrainant le décès de la patiente au service de réanimation.

**Consentement de la famille:** suite à l'évolution létale de la patiente; sur la base de nos explications portant sur l'intérêt de la science concernant cette pathologie; nous avons reçu le consentement de la famille pour la publication.

## Discussion

**Discussion scientifique sur les points forts et les limites associées à ce rapport de cas:** ce cas d'endocardite de Libman-Sacks est le premier diagnostiqué comme tel dans notre service. Très peu de cas sont rapportés dans la littérature alors que la prise en charge est un vrai défi pour le praticien. Le retard d'évocation du diagnostic et l'absence de certains tests diagnostiques dans notre structure constituent les limites de ce rapport de cas.

### Discussion de la littérature médicale pertinente, avec références

L'endocardite de Libman-Sacks constitue une des manifestations cardiaques du lupus systémique. Il s'agit d'une endocardite non infectieuse. Elle touche surtout les valves mitrales puis aortiques, mais toutes les quatre valves et toute la surface de l'endocarde peuvent être touchées. Les végétations sont de petites tailles comme dans notre observation et passent souvent inaperçues à l'échographie transthoracique, mais des végétations plus importantes peuvent se voir. Roldan *et al*. ont trouvé une prévalence de 43% d'endocardite de Libman-Sacks dans une série de 69 patients lupiques par la réalisation systématique d'une échographie transoesophagienne. Pour d'autres auteurs, l'endocardite de Libman-Sacks représente moins de 10% des patients atteints de lupus. Cette différence de prévalence s'explique probablement par l'absence de réalisation systématique d'échographie cardiaque transoesophagienne. La présence d'un syndrome des antiphospholipides serait un facteur favorisant la formation de végétations [2]; ceci a été retrouvé dans notre cas. La pathogenèse de l'endocardite de Libman-Sacks impliquerait la formation d'un thrombus sur une valve endommagée par les dépôts de complexes immuns, induisant une inflammation, qui évolue vers une fibrose avec distorsion et dysfonction [3].

L'examen anatomopathologique des végétations montre des dépôts de fibrine, un infiltrat de cellules inflammatoires mononucléées, de la fibrose, des néovaisseaux et parfois des dépôts d'immunoglobulines et de complément [2]. Dans notre cas, la patiente n'ayant pas été opérée, nous n'avons pas eu de résultat anatomopathologique. Il n'existe pas de protocole thérapeutique consensuel dans la littérature, d'où l'intérêt des réunions de concertation pluridisciplinaire au sein desquelles la place de l'interniste est primordiale. De nombreuses études révèlent que l'utilisation de corticoïdes et d'immunosuppresseurs semble n'avoir aucun effet sur la régression des lésions valvulaires [4].

Selon d'autres études, les corticoïdes accélèrent la cicatrisation de la lésion myocardique auto-immune en réduisant l'inflammation et l'activité de la maladie, mais peut aggraver une infection bactérienne et sont donc non recommandées en cas de suspicion d'endocardite infectieuse [5] ou de surinfection bactérienne. Dans le cas de notre patiente, l'issue a été fatale après introduction de la corticothérapie malgré une antibiothérapie bien menée sur plus d'une dizaine de jours sans amélioration clinique. En ce qui concerne l'anticoagulation, elle s'impose à titre préventif lorsque le risque thromboembolique est élevé. En effet, la présence d'atteinte valvulaire dans un contexte de Syndrome des antiphospholipides (SAPL), est associée à un risque élevé de survenue de thromboses artérielles et d'évènements emboliques cérébraux. La survenue de complications hémorragiques peut toutefois en limiter la prescription et donc exposer les patients à plus de risque thromboembolique. L'anticoagulation par anti-vitamine K est le traitement de référence avec un INR cible entre 3 et 3,5. Elle est parfois associée à une chirurgie valvulaire. Les anticoagulants oraux directs semblent ne pas être efficaces dans cette indication [5,6]. Chez notre patiente, l'anticoagulation n'a pas été introduite, du fait de la réception tardive des résultats à la recherche des Anticorps anti-phospholipides. Selon Lahatriniavo Ramiandrisoa *et al*. l'adjonction des antipaludéens de synthèse permettrait d'améliorer le pronostic de ces patients [2].

En cas de dysfonction valvulaire avec retentissement hémodynamique important, le contrôle peut se faire par un traitement conservateur: immunosuppresseurs, anticoagulants, et le traitement spécifique de l'insuffisance cardiaque (diurétiques, bêtabloqueurs et IEC). Si le tableau est sévère et réfractaire, la correction chirurgicale s'impose. Le SAPL est la cause auto-immune la plus fréquente d'endocardite non infectieuse ayant nécessité un remplacement valvulaire [7]. La plastie est préférée chaque fois qu'elle est possible. Quand le remplacement valvulaire est inévitable, le choix entre une bio prothèse ou une prothèse mécanique dépendra de l'âge du patient, de ses souhaits et, chez la femme, du désir de procréer. Il est à rappeler qu'en cas de ELS, le risque de calcification de la bio prothèse doit être pris en considération et l'anticoagulation peut s'avérer nécessaire. De ce fait, une prothèse mécanique peut assurer de meilleurs résultats pour les valvulopathies mitrales dues à l'endocardite de Libman-Sacks [5,8].

### Justification scientifique de toute conclusion (y compris l'évaluation des causes possibles)

Le diagnostic différentiel morphologique se pose avec un myxome, un rhabdomyome, et autres masses pédiculées valvulaires [3,5]. Toutefois, le diagnostic différentiel entre endocardite de Libman-Sacks et endocardite infectieuse est obligatoire. Dans cet aspect, trois données de laboratoires sont importantes: numération leucocytaire, taux de CRP et les cultures de sang. Les leucocytes ont tendance à diminuer au cours de l'activité lupique, ce qui n'est pas le cas dans l'endocardite infectieuse. Un taux de CRP élevé suggère une cause infectieuse, comme les patients lupiques sont moins capables de présenter une réponse exubérante de cette protéine. Cependant, pour trancher le diagnostic, les hémocultures sont primordiales [1]. Concernant notre cas, nous avions objectivé une leucopénie, une CRP élevée avec des séries d'hémocultures négatives.

## Conclusion

Le diagnostic d'endocardite non infectieuse doit être évoqué quand l'évolution d'une endocardite d'allure infectieuse est atypique sous antibiothérapie. Malgré quelques similitudes cliniques et échocardiographiques, l'endocardite de Libman-Sacks est une entité dont l'évolution peut être fatale quand le diagnostic et surtout le traitement n'est pas instauré précocement. Il faut savoir l'évoquer chez des patients souffrant de pathologies fréquemment associées à un SAPL (ELS, SEP) et craindre la surinfection bactérienne. La prise en charge doit être multidisciplinaire, associant cardiologues et internistes. Des études plus approfondies devraient être réalisées afin d'établir un protocole thérapeutique consensuel insistant sur la place de la corticothérapie et des antipaludéens de synthèse dans la prise en charge de ces patients.
